# Analysis of Occupational Balance and Its Relation to Problematic Internet Use in University Occupational Therapy Students

**DOI:** 10.3390/healthcare9020197

**Published:** 2021-02-11

**Authors:** Alejandro Romero-Tébar, Marta Rodríguez-Hernández, Antonio Segura-Fragoso, Pablo A. Cantero-Garlito

**Affiliations:** 1 Faculty of Health Sciences, University of Castilla La Mancha, 45600 Talavera de la Reina, Spain; alejandroromero.to@hotmail.com (A.R.-T.); Antonio.Segura@uclm.es (A.S.-F.); Pablo.Cantero@uclm.es (P.A.C.-G.); 2 Occupational Therapy Department, Residence Ntra. Sra. de la Piedad, 45800 Quintanar de la Orden, Spain

**Keywords:** occupational balance, internet addiction, problematic internet use, phubbing, university students

## Abstract

(1) Objective: to explore and describe the relationship between the occupational balance of university students taking a Degree in Occupational Therapy and the problematic use of the Internet and how these, in turn, favour or not the appearance of phubbing behaviour which involves “a behaviour that happens when an individual looks at his mobile phone during a conversation with other individuals, escaping from interpersonal communication”. (2) Methods: this is a quantitative descriptive study of an observational and cross-sectional nature, not experimental. The Spanish version of the Occupational Balance Questionnaire (OBQ-E), the Internet Addiction Test and the Phubbing Scale were used for data collection. 192 university students taking the Degree in Occupational Therapy of the University of Castilla–La Mancha participated (168 women and 24 men). (3) Results: the average score obtained in the OBQ-E was 38.7, indicating a moderate occupational balance; and (4) Conclusions: occupational therapy students from the Faculty of Health Sciences of the University of Castilla–La Mancha have a moderate occupational balance. Furthermore, this is negatively related to both problematic Internet use and phubbing, so a higher occupational balance indicates less Internet addiction and less phubbing.

## 1. Introduction

### 1.1. Occupational Balance

The concept of occupational balance (OB) has been present since the beginning of occupational therapy. The term first appeared in 1997 [1] although the first reference to the study of balance between occupations dates from 1910 [2], which contemplates 24 h divided into changing periods of work, rest and recreation. On the other hand, among the most recognized authors we find Ann A. Wilcock, who highlights that occupational balance has a direct benefit on perceived health [3]. It is defined as “the individual’s subjective experience of having the ‘right mix’ of occupations in their occupational pattern” [4]. Occupational balance has a personal, subjective and particular character which is composed of two dimensions, one quantitative (amount of time invested and amount of occupations performed) and one qualitative (variety of occupations performed and satisfaction with them and the time spent in them) [5]. This is a multidimensional concept which is influenced by various factors, such as satisfaction with the occupations, time spent in them, the characteristics of the occupations performed (physical, mental, social and rest), the occupational pattern and roles, and the daily rhythm of the occupations [4].

OB has been studied in populations with different characteristics. For example, in a healthy population, having a good OB appears to be a predictor of health in women [6]. Furthermore, occupational balance has significant associations with life satisfaction [7]. Another area that has been emphasized in the study of occupational balance is retirement, where the main findings identify a pattern that involves the passage from an occupational imbalance caused by work demands to an imbalance related to the loss of occupational commitments [8]. Studies on the implications of OB in different pathologies have also been carried out, showing that, for example, in people with neurological pathology, alterations in OB are experienced, reducing mainly participation in work [9]. In the case of people with mental illness, it has been found that feeling competent and having a balance between different significant occupations helps the person to control the illness [10].

However, there is little research analysing OB in university students. In one study [11], students thought that evaluating their OB allowed them to become aware of how they organised their daily routine, which in turn led them to look for ways to achieve this, such as prioritising the fulfilment of some needs over others. We would like to highlight a recent study with 219 Spanish students. The findings reveal that a higher OB indicates better physical, mental and global health and a higher health-related quality of life, and that students with higher OB perform better academically [5]. Finally, in a study conducted with 87 undergraduate students studying occupational therapy at the University of Quilmes in Argentina, 62% felt dissatisfied with their daily routine [12].

### 1.2. Internet Addiction

On the other hand, information and communication technologies have generated great changes and innovations that have influenced young people’s daily lives, as they have incorporated them on a regular basis [13]. This fact has led to increased concern about the existence of Internet addiction [14]. In this sense [15] states that psychosocial stressors such as insomnia, depression, stress and low self-esteem can occur. In addition, a recent study [16] suggests that Internet addiction was associated with poor eating habits and problems with sleep and fatigue.

As far as university students are concerned, in a study [17] with 162 participants from the United Kingdom taking different degrees, it was found that problematic use of the Internet is negatively associated with aspects such as motivation to study or self-efficacy in learning. In no case has the relationship to the occupational balance of the students been studied. In this sense, it should be noted that boredom during free time contributes to Internet addiction [18].

Related to this phenomenon is the addiction to social networks. This is defined by Andreassen [19] as “being too preoccupied with social networking sites, being driven by a strong motivation to log in or use social networks and devoting so much time and effort to it that it impairs other social activities, studies/work, interpersonal relationships, and/or psychological health and well-being”. Under this perspective, Giunchiglia [20] showed that the use of social networks during academic activities reflected a negative association with students’ academic performance.

On the other hand, we should consider the so-called phubbing behaviours, which involve the act of ignoring a person and one’s environment in order to focus attention on the mobile device, tablet, or any technological device. Phubbing can be described as “a behaviour that happens when an individual looks at his mobile phone during a conversation with other individuals, escaping from interpersonal communication” [21].

These authors highlight the multidimensional nature of the term. These dimensions involve: mobile phone addiction, Internet addiction, social network addiction and gambling addiction. This set implies the use of a certain amount of time on the mobile device; in this sense, some authors who have studied the use and time spent on the mobile phone by university students [14,22] find that the most frequent activities on the Internet are those related to e-mail and sending messages through social networks and listening to music, and those in which they spend less time are in gambling and adult websites.

One of the main effects of this behaviour lies in social interaction. Thus, the effects that phubbing has on social interaction have been studied, where findings reveal that the experience of phubbing in a conversation has a negative impact on the quality of perceived communication and satisfaction with it [23]. Furthermore, despite the few studies concerning this behaviour, some authors consider that being busy with mobile phones during lessons is an act of phubbing [24].

The aim of this study is to explore and describe the relationship between the occupational balance of university students taking the Degree in Occupational Therapy and the excessive use of the Internet, and how these, in turn, favour or not the appearance of phubbing behaviour. The specific objectives pursued are to know the occupational balance of university students taking the Degree in Occupational Therapy, to determine the level of Internet addiction of university students taking the Degree in Occupational Therapy, and to establish the incidence of phubbing behaviour in university students on occupational therapy courses.

## 2. Materials and Methods

### 2.1. Design

Descriptive observational study, which aims to analyse the results obtained in a sample of 192 students enrolled in the Degree in Occupational Therapy of the Faculty of Health Sciences of Talavera de la Reina (University of Castilla–La Mancha).

### 2.2. Participants

The sample was made up of students enrolled in the four courses of the Degree in Occupational Therapy (Talavera de la Reina, University of Castilla La Mancha). For the estimation of the sample size, a correlation coefficient was used, accepting an alpha risk of 0.05 and a beta risk of 0.2 in bilateral contrast. According to the calculations carried out, the project would assume a minimum of 85 students, with a correlation coefficient of −0.2. Estimating a 70% response, the sample size was 192 enrolled students. For the recruitment of participants, permission was granted by the Dean of the Faculty of Health Sciences.

The questionnaire was administered at the beginning of classes, in each of the four courses of the degree. As criteria for inclusion, participants had to be enrolled in the Degree in Occupational Therapy at the Faculty of Health Sciences in Talavera de la Reina (University of Castilla–La Mancha), be between 18 and 65 years old and voluntarily participate in the study, previously filling in the informed consent form. Students who did not attend classes during the administration of the questionnaire or who were doing international internships were excluded.

### 2.3. Instrument

Three assessment scales were administered to address different aspects of the study. This questionnaire consisted, firstly, of 5 items related to socio-demographic data.

#### 2.3.1. Occupational Balance Questionnaire (OBQ-E)

The study consisted of 13 items included in the Spanish version of the Occupational Balance Questionnaire (OBQ-E) [5], which explore the balance between different types of activity, the significance and time attributed to them, and perceived satisfaction, using a 6-point Likert-type response scale, from 0 (completely disagree) to 6 (completely agree). The final score of the questionnaire ranges from 0 to 65 points where a higher score indicates a better occupational balance.

#### 2.3.2. Internet Addiction Test (IAT)

The Internet Addiction Test (IAT) [25], is an instrument for assessing the symptoms of Internet addiction consisting of 20 items that are answered according to a 5-point Likert scale where 1 (never) and 5 (always). The higher the score, the higher the level of Internet addiction.

The IAT score suggests that, between 20 and 39 points, users are considered to have full control over their Internet use. Between 40 and 69 points, users are considered to be experiencing occasional or frequent problems due to Internet use. Finally, a score between 70 and 100 indicates that they have significant problems caused by Internet use.

#### 2.3.3. The Phubbing Scale (TPS) 

The Phubbing Scale [26] consists of 10 items graded on a five-point Likert scale from 1 (never) to 5 (always). The score ranges from 1 to 50 points and includes 2 factors: “communication disturbance” and “telephone obsession” where higher scores indicate that participants make communication difficult by being with their mobile phone in a face-to-face environment and that they constantly need their mobile phone, respectively.

### 2.4. Statistical Analysis

Once the data collection work was completed, all the information gathered was processed, with the aim of systematizing it and making it understandable. Of the various methods available for this purpose, and taking into account the size of the sample, the number of variables and the resources available, digital processing was chosen. Tabular sheets with the data collected were used to proceed with the analysis of the results, placing in the columns the numerical values relating to the instruments and in the rows the subjects that made up the sample (n = 192).

In this work, it was decided to present the information obtained through tables, which reflect the percentage of students according to age, sex and course. Descriptive statistics were used to analyse the differences in the socio-demographic variables and in the items of the instruments used: means and standard deviations. The statistical significance of the differences between proportions was analysed using the Chi-square test, and analysis of variance (ANOVA) was used for the difference between means. The analysis of the association between the instruments was carried out using the Pearson correlation coefficient.

The data were analysed with the IBM SPSS Statistics package (version 24.0 SPSS for Windows; SPSS Inc., Chicago, IL, USA).

### 2.5. Ethical and Legal Considerations

The participants were informed about the fundamental aspects and objectives of the project at the beginning of the questionnaire. It also included informed consent and the possibility of withdrawing their participation at any time. In carrying out the study, compliance with the Good Clinical Practice Guidelines and the recommendations of the ethical principles of the Declaration of Helsinki were taken into account, and the confidentiality of participants’ data was paramount. All of this information complied with Organic Law 3/2018, of 5 December, on the protection of personal data and the guarantee of digital rights. Finally, the Clinical Research Ethics Committee of the Integrated Area of Talavera de la Reina 49/2019 has issued a favourable opinion.

## 3. Results

### 3.1. Socio-Demographic Characteristics of the Participants

In Table 1, the percentages by sex, age and course of the sample of 192 students enrolled in the Degree in Occupational Therapy (Faculty of Health Sciences) are presented. 87.5% of the sample was composed of women and 35.9% was in the age range of 20 to 21 years.

### 3.2. Analysis of the Occupational Balance: OBQ-E Instrument According to Socio-Demographic Data

The average score obtained on the OBQ-E instrument was 38.7 points. Considering that the score ranges from 0 to 65 points on this instrument, the results shown in Table 2 may indicate that the students in the sample do not have a high occupational balance.

In relation to the differences shown in the variable ‘course’, it is observed that the average in the score of the instrument increases as the student evolves from course to course, being statistically significant (*p* = 0.012).

### 3.3. Analysis of the Values Obtained in the Items of the OBQ-E Intrument

Table 3 shows the scores obtained on the different items of the OBQ-E instrument. The averages range from 2.52 to 3.86 and are, therefore, in the following response categories: (2) ‘strongly disagree’ and (3) ‘somewhat disagree’. The category ‘strongly disagree’ was assigned 0 points, while the category ‘strongly agree’ was assigned a score of 5.

### 3.4. Analysis of the Internet Addiction of the Selected Sample (IAT Instrument)

The average score on the IAT instrument was 49.2 points. No significant differences were found between the averages according to age, grade and sex. However, male students recorded higher scores than female students. 85.9% of the sample obtained a total score between 40 and 69 points, with a minority (3.2%) obtaining scores below 39. The remaining 11.0% recorded scores above 70 points.

### 3.5. Analysis of the Values Obtained in the Items of the IAT Instrument

The scores obtained in the sample were analysed according to socio-demographic characteristics. Statistically significant differences were found in items 1, 2, 4 and 13 (*p* = 0.048; *p* = 0.002; *p* = 0.002 and *p* = 0.026, respectively). Table 4 shows the mean values obtained in each of the items that make up the IAT instrument.

### 3.6. Analysis of Data Obtained on The Phubbing Scale

The average score on The Phubbing Scale was 25.8 points, with the total score on the scale being 50 points. Homogeneous phubbing behaviour was observed in all participants.

### 3.7. Analysis of the Values Obtained in the Items of The Phubbing Scale

The scores obtained in the sample were analysed according to socio-demographic characteristics. Statistically significant differences were found in items 2, 6, 7 and 10 (*p* = 0.022; *p* = 0.041; *p* = 0.002 and *p* = 0.047, respectively). Table 5 shows the average values obtained in each of the items that make up the scale.

### 3.8. Analysis of the Correlation of the Instrument Used

The results show that the OBQ-E instrument has a low and negative correlation with the IAT (*p* = 0.001) and TPS (*p* = 0.013) instruments and does not correlate with the ‘age’ variable. The IAT instrument shows a positive and very strong correlation with the TPS instrument (*p* < 0.001), and low and negative correlation with the ‘age’ variable (*p* = 0.026). The TPS instrument is not correlated with age (Table 6).

## 4. Discussion

The aim of this paper was to explore the OB of students taking the Degree in Occupational Therapy at the Faculty of Health Sciences of the University of Castilla–La Mancha and its possible relationship with other factors such as Internet addiction and phubbing behaviour.

The average score obtained from the OBQ-E was 38.7. Taking into account that the scale scores from 0 to 65, it can be said that this is a moderate score, which reflects a certain imbalance between different activities of daily life (work, study, household tasks, leisure, rest and sleep) or between those that give energy and those that take away energy, as well as the significance, satisfaction and time attributed to these.

There is a significant relationship between the OB and the academic year, so that, as academic years are passed, a greater occupational balance appears. This may be due to the fact that, as students’ progress through the degree, they have acquired strategies to cope with the demands of the different subjects. In the first year, students have just come from high school, from an educational model that is much more accompanied than that offered by the university. This change requires adaptation to new forms of teaching and learning that in the first months may require a high level of adaptation. This demand can be moderated over time at the university. This is somewhat more explicit in a study carried out with university students of occupational therapy for whom knowing their OB allowed them to become aware of how they organized their daily routine and led them to seek strategies to achieve it [11].

Our results show that students have a lower level of OB than the data obtained in a study conducted with university students of occupational therapy [5]. Furthermore, in this same study, no correlation was found between OBQ-E and academic year. On the other hand, the results of our sample coincide with this other study, in terms of the mean obtained by men (40), which was higher than that obtained by women.

Another study, which aims to introduce the questionnaire as an instrument for measuring OB [4], explores the OB of 67 Swedish adults and obtained an average OBQ score of 43.5. This is higher than the score obtained in our sample. The scores may be due to the fact that in both cases the sample consists of healthy adults, and the difference between scores may be due to the students’ familiarity with the concept.

It was expected that students with higher OB would have less addiction to the Internet. As the results of this work reveal, there is a negative relationship between OB and Internet addiction, so the higher the OB, the less Internet addiction. The results showed that 3.2% of the sample scored between 20–39 points, where users are considered to have full control over their Internet use. The majority of students in the sample scored between 40–69 points (85.9%), indicating that these users may experience occasional or frequent problems due to their Internet use. The remaining 11% were students who scored above 70 points, corresponding to those who had significant problems caused by their Internet use.

In our sample of students, although men obtained higher average scores, we found no significant associations with socio-demographic data. Thus, there are studies that similarly find no significant differences that related internet addiction to gender [27], and yet others argue that male sex is a predictor of internet addiction [15,28,29].

The results of this study revealed that internet addiction was significantly correlated with phubbing among the students in the sample. Evidence relating to phubbing is very limited and has never been associated with OB. However, a study by Ivanova [30] also found a positive correlation between mobile phone addiction and phubbing. This same study revealed that gender did not moderate the relationship between mobile phone addiction and phubbing, which is consistent with the findings of this paper and that of Chotpitayasunondh [23].

Previous studies obtained contradictory results: Karadag [21] found that gender moderated the relationship between mobile phone addiction and phubbing (the relationship was stronger for women than for men). This idea also differs from the results found in our study, as it was men who recorded higher scores.

This work has different limitations. Firstly, the sample was reduced to university students of the same Degree from the same Faculty so their characteristics are similar. It would be advisable to be able to extend the sample to university students belonging to other degrees and/or from other localities. Therefore, our suggestion for future research is to analyse a sample representing a wider adult age range to see how age may or may not moderate the relationships studied.

Secondly, the fact that the participants in the sample are in contact with the concept of occupational balance may condition the scores obtained in the questionnaire. Therefore, extending the sample to students from other degree programmes could affirm the existence of this relationship.

Finally, the study followed a cross-sectional design, which means that the results obtained cannot infer a cause-effect relationship. It would be relevant to be able to carry out a longitudinal study to analyse more closely the relationship between OB and Internet addiction, together with phubbing, as well as a qualitative study to find out what could be the reasons why more time is spent on Internet use, phubbing and why the occupational balance decreases.

## 5. Conclusions

Students of occupational therapy at the Faculty of Health Sciences of the University of Castilla–La Mancha have a moderate occupational balance. This is independent of age and sex, but correlates positively with the academic year, with students in higher education obtaining better OB.

Furthermore, students’ OB is negatively related to both problematic Internet use and phubbing, so a higher OB indicates less Internet addiction and less phubbing. In this sense, internet addiction showed a strong positive correlation with phubbing, so that the higher the internet addiction, the higher the phubbing of students.

According to the results of the study, it can be stated that most students can present occasional problems due to Internet use, and that the greater the Internet use, the greater the phubbing behaviour and attitudes.

## Figures and Tables

**Table 1:** Sociodemographic characteristics of the participants (n = 192).

Sociodemographic Characteristics	n	%
**Sex**		
Man	24	12.5
Woman	168	87.5
**Course**		
First	54	28.1
Second	50	26.0
Third	44	22.9
Fourth	44	22.9
**Age**		
From 18 to 19 years old	55	28.6
From 20 to 21 years old	69	35.9
From 22 to 23 years old	37	19.3
24 years of age or older	31	16.1

**Table 2:** Analysis of the occupational balance: OBQ-E according to sociodemographic data.

Sociodemographic Data	n	Average (SD)	*p*-Value
**Sex**			*0.472*
Man	24	40.0 (12.9)	
Woman	168	38.5 (9.0)	
**Course**			*0.012*
First	54	36.7 (9.6)	
Second	50	37.4 (8.3)	
Third	44	38.5 (9.7)	
Fourth	44	42.7 (9.9)	
**Age**			*0.126*
From 18 to 19 years old	55	36.9 (10.2)	
From 20 to 21 years old	69	40.8 (8.7)	
From 22 to 23 years old	37	38.1 (10.9)	
24 years of age or older	31	37.8 (8.0)	

**Table 3:** Average values obtained in the items of the OBQ-E instrument (n = 192).

Occupational Balance Questionnaire-E	Average (SD)
1. I have a balance between the things I do for others and the things I do for myself.	2.8 (1.2)
2. The activities I do in my daily life make sense to me.	3.8 (0.9)
3. I make sure I do the things I really want to do.	3.5 (0.9)
4. I maintain a balance between the different activities of my daily life (work, study, housework, leisure, rest and sleep).	2.6 (1.1)
5. I have enough variety between activities I do alone and those I do with others.	3.1 (1.1)
6. If I think about a normal week, I have enough things to do.	3.9 (1.0)
7. I have enough time to do the things I need to do.	2.8 (1.1)
8. I maintain a balance between physical, social, intellectual and rest activities.	2.5 (1.2)
9. I am satisfied with the time I spend on the different activities in my daily life.	2.8 (1.1)
10. If I think about a normal week, I am satisfied with the amount of activities I do.	2.9 (1.1)
11. I have enough variety between the things I have to do and the things I want to do.	2.8 (1.1)
12. I have a balance between the different activities that give energy and the activities that take away energy.	2.5 (1.1)
13. I am satisfied with the time I spend on rest, recovery and sleep.	2.6 (1.3)

**Table 4:** Average values obtained in the items of the IAT instrument (n = 192).

Internet Addiction Test	Average (SD)
1. How often do you connect to the Internet more than you expect?	3.9 (0.9)
2. How often do you neglect household activities to stay more connected?	2.4 (1.1)
3. How often do you prefer the excitement of being connected to your partner or the direct relationship with your friends?	2.3 (1.4)
4. How often do you form new relationships with users on the Internet?	1.8 (0.9)
5. How often do people close to you complain about the amount of time you spend online?	2.1 (1.1)
6. How often are your grades or academic activities negatively affected by the amount of time you spend on the Internet?	2.1 (1.1)
7. How often do you check your email before you do another task you need to do?	2.5 (1.2)
8. How often does time spent on the Internet negatively affect your performance or productivity at work?	2.3 (1.0)
9. How often are you defensive or secretive when someone asks you what you do on the Internet?	1.8 (1.0)
10. How often do you block out unpleasant thoughts from your life with pleasant thoughts related to the Internet?	2.2 (1.1)
11. How often do you anticipate when you will be online again?	2.1 (1.2)
12. How often do you fear that life without the Internet would be boring, empty or sad?	2.3 (1.2)
13. How often do you get angry if someone disturbs you while you are online?	1.8 (1.0)
14. How often do you lose sleep because you go online during the night?	2.0 (1.1)
15. How often do you feel worried about not being connected or imagine being connected?	1.6 (0.9)
16. How often do you say: “a few more minutes”, when you are connected?	2.7 (1.3)
17. How often do you try to reduce the time you spend on the Internet and fail to do so?	2.4 (1.1)
18. How often do you try to hide the time you are online?	1.7 (1.0)
19. How often do you spend more time on the Internet than on dating?	1.7 (0.9)
20. How often do you feel depressed, moody or nervous when you are not connected, but feel better when you are connected again?	1.6 (0.9)

**Table 5:** Average values obtained in the items of The Phubbing Scale (n = 192).

The Phubbing Scale	Average (SD)
1. I keep an eye on my mobile phone when I am in the company of other people.	2.8 (1.1)
2. I am busy with my mobile phone when I am with my friends.	2.1 (0.9)
3. Other people complain about my use of the mobile phone.	1.8 (0.9)
4. I am busy with my mobile phone when I am with my relatives.	2.2 (1.0)
5. My partner is annoyed that I am busy with my mobile phone (family if you don’t have a partner).	1.9 (1.1)
6. My mobile phone is within my reach.	4.3 (0.8)
7. The first thing I do when I wake up is to look at my mobile phone messages.	4.0 (1.2)
8. I feel empty without my mobile phone.	2.4 (1.2)
9. My mobile phone usage is increasing every day.	2.2 (1.1)
10. The time I spend on social, personal or professional activities is reduced by the time I use my mobile phone.	2.1 (1.1)

**Table 6:** Correlation of the instruments used.

Correlations		IAT	TPS	Age
OBQ-E	Pearson Correlation	−0.238	−0.179	−0.091
	*p*-value	*0.001*	*0.013*	*0.211*
IAT	Pearson Correlation		0.767	−0.160
	*p*-value		*0.000*	*0.026*
TPS	Pearson Correlation			−0.096
	*p*-value			*0.183*

*p*-values of the correlation. IAT: Internet Addiction Test. TPS: The Phubbing Scale.
